# D-alanine synthesis and exogenous alanine affect the antimicrobial susceptibility of *Staphylococcus aureus*

**DOI:** 10.1128/aac.01936-24

**Published:** 2025-06-12

**Authors:** Yujin Suzuki, Miki Kawada-Matsuo, Vy Ton That Thuan, Mi Nguyen-Tra Le, Takemasa Sakaguchi, Hitoshi Komatsuzawa

**Affiliations:** 1 Department of Bacteriology, Hiroshima University Graduate School of Biomedical and Health Sciences, Hiroshima, Japan; 2 Department of Virology, Hiroshima University Graduate School of Biomedical and Health Sciences 592299 , Hiroshima, Japan; 3 Project Research Centre for Nosocomial Infectious Diseases, Hiroshima University, Hiroshima, Japan; The Peter Doherty Institute for Infection and Immunity, Melbourne, Victoria, Australia

**Keywords:** *Staphylococcus aureus*, MRSA, D-alanine

## Abstract

D-alanine is an important amino acid for peptidoglycan biosynthesis in *Staphylococcus aureus*. In addition, D-alanine is used for the modification of teichoic acids to weaken the net surface negative charge, leading to decreased susceptibility to cationic antimicrobial agents. D-alanine synthesis is dependent on only two enzymes. One is alanine racemase, encoded by the *alr1* gene, which reversibly converts L-alanine and D-alanine. The other is D-amino acid transaminase, encoded by the *dat* gene, which synthesizes D-amino acids from α-keto acids and other D-amino acids. In addition, the uptake of L- and D-alanine is dependent on the alanine transporter CycA. To reveal the relationship between D-alanine supply and antimicrobial susceptibility, we evaluated antimicrobial susceptibility in *alr1, dat,* and *cycA* inactivation mutants. These mutants, especially the Δ*alr1* and Δ*cycA* mutants, presented increased susceptibility to β-lactams, D-cycloserine, bacitracin, lysostaphin, and cationic antimicrobial agents such as aminoglycosides, nisin A, and daptomycin. The net negative charge of the cell surface increased in the Δ*alr1* and Δ*cycA* mutants. The changes in susceptibility to antimicrobial agents and cell surface charge were restored in their gene-complemented mutants. Furthermore, in an alanine-depleted medium, the MIC for oxacillin decreased significantly, and the MIC for gentamicin also decreased slightly. Clinical MRSA strains also showed significantly increased susceptibility to oxacillin in the alanine-depleted medium. These results indicate that D-alanine deficiency leads to impaired peptidoglycan and increased net surface negative charge, resulting in increased antimicrobial susceptibility.

## INTRODUCTION

D-alanine is one of the amino acids used in the cell wall of most bacteria, including the opportunistic pathogen *Staphylococcus aureus* (1). The terminal D-alanyl-D-alanine (D-ala-D-ala) of the peptidoglycan pentapeptide plays an important role in the formation of peptidoglycan crosslinks catalyzed by penicillin-binding proteins (PBPs) (2). Since PBPs are the target of β-lactams, and peptidoglycan D-ala-D-ala is the target of glycopeptide antibiotics, changes in the amount of intracellular D-ala-D-ala and its precursor D-alanine may be associated with the antimicrobial effect of these two classes of antibiotics. In addition, D-alanylation of teichoic acids in Gram-positive bacteria is known to weaken the negative charge of bacterial cells, as teichoic acids are negatively charged (3, 4). This system is dependent on DltABCD proteins, and the *dltA*-deficient strain is known to exhibit a high susceptibility to cationic antimicrobials (4).

**Fig 1:**
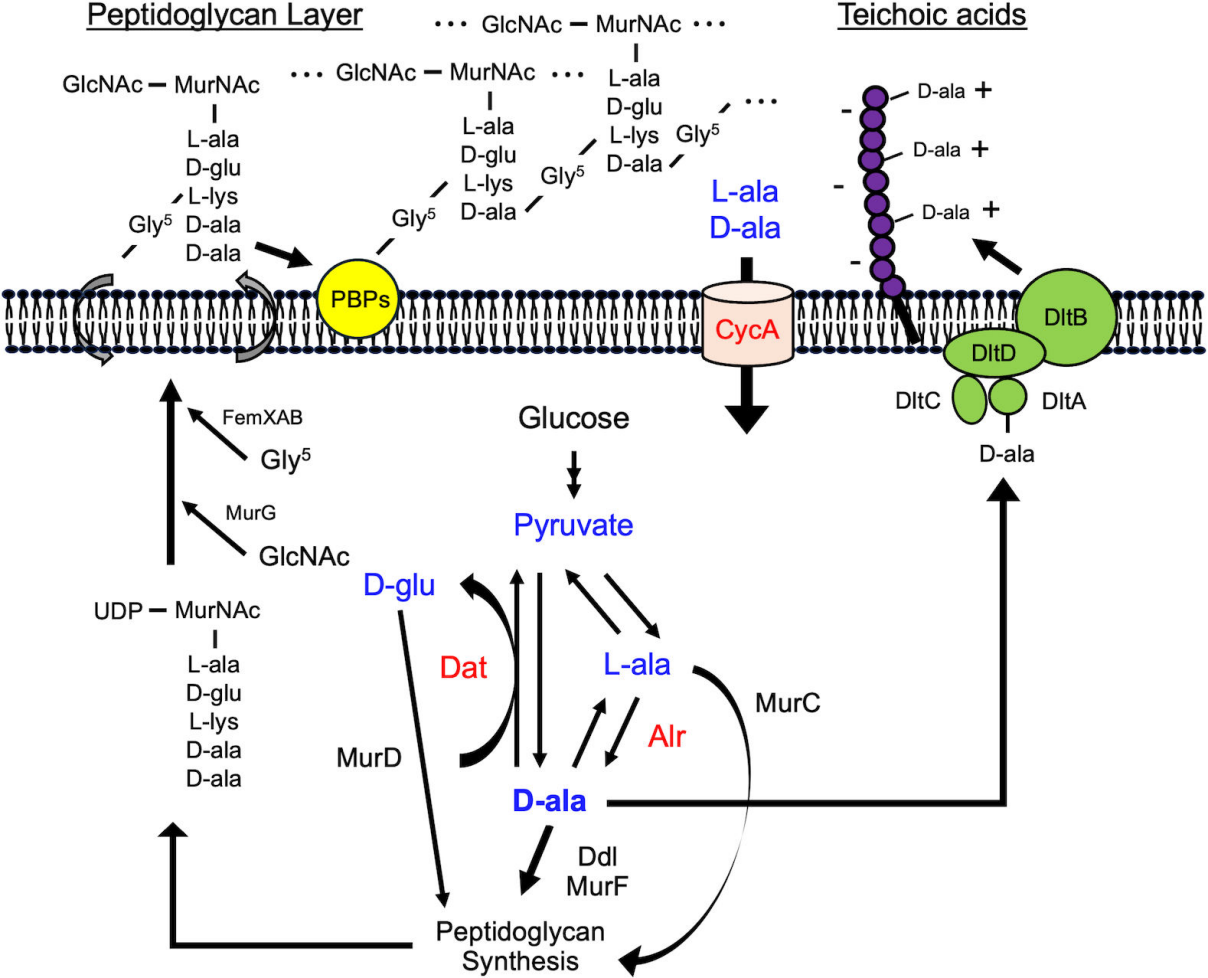
Graphical summary of D-alanine synthesis and cell wall utilization in *S. aureus*. There are only two enzymes involved in the synthesis of D-alanine in *S. aureus*. Alanine racemase (Alr) reversibly converts L-alanine and D-alanine, and D-amino acid transaminase (Dat) synthesizes D-alanine from pyruvate and amino groups obtained from other D-amino acids, such as D-glutamate. In addition, the alanine transporter CycA imports L- and D-alanine from the exogenous environment. D-alanine is used for the synthesis of peptidoglycan via D-alanine-D-alanine ligase (Ddl) and UDP-N-acetylmuramoyl-tripeptide-D-alanyl-D-alanine ligase (MurF). L-alanine and D-glutamate are also necessary for the synthesis of peptidoglycan. The terminal D-ala-D-ala in the peptidoglycan pentapeptide is necessary for the transpeptidase activity of PBPs. On the other hand, the Dlt system also requires D-alanine for the modification of teichoic acids, which regulates the net charge of the cell surface. GlcNAc: N-acetylglucosamine, MurNAc: N-acetylmuramic acid, Gly^5^: pentaglycine.

On the other hand, there are only two enzymes for the synthesis of D-alanine in *S. aureus* (Fig. 1) (5, 6). One is alanine racemase (Alr), encoded by the *alr1* and *alr2* genes, which reversibly converts L-alanine and D-alanine. To date, only the *alr1* gene has been characterized for its function in *S. aureus* (7), and Alr1 is thought to be the major enzyme for D-alanine production (6). Another pathway involves the D-amino acid transaminase (Dat), encoded by the *dat* gene, which converts α-keto acid into D-amino acids with an amino group obtained from other D-amino acids (8). This enzyme is usually involved in the synthesis of D-glutamate rather than D-alanine, but when alanine racemase is absent, it synthesizes D-alanine (6). Since an *alr1* and *dat* double-deficient strain was reported to exhibit D-alanine auxotrophy (5, 6), these two genes seem to be responsible for D-alanine synthesis. In addition to these systems, *S. aureus* has an alanine transporter, CycA (also known as AapA), which imports exogenous alanine (9, 10). It has been reported that CycA only imports alanine and that the absence of CycA improves susceptibility to β-lactams in methicillin-resistant *S. aureus* (MRSA) (9).

In this study, we verified the comprehensive relationship between D-alanine supply and antimicrobial susceptibility by examining the antimicrobial susceptibility of *alr1-, dat-,* and *cycA*-deficient mutants and their gene-complemented mutants. We also verified the effect of exogenous alanine on antimicrobial susceptibility.

## RESULTS

### Antimicrobial susceptibility of the Δ*alr1*, Δ*dat*, and Δ*cycA* mutants

First, the susceptibility of strains deficient in factors involved in D-alanine synthesis (*alr1, dat*) and uptake (*cycA*) to several antimicrobial agents was analyzed by minimum inhibitory concentration (MIC) assays and disk tests. We used the *S. aureus* MW2 strain to construct genetic mutants for susceptibility tests. MW2 is known as a community-acquired MRSA that carries only *blaZ* and *mecA* for antimicrobial resistance genes, resulting in susceptibility to most antimicrobial agents except β-lactams (11). The MICs of oxacillin were decreased in the *alr1*-inactivated (Δ*alr1*) mutant, *dat*-inactivated (Δ*dat*) mutant, and *cycA*-inactivated (Δ*cycA*) mutant compared with the wild type (WT); particularly, the significant changes were observed in the Δ*alr1* and Δ*cycA* mutants (Table 1). The MICs of cefazolin were decreased in the Δ*alr1* and Δ*cycA* mutants. The MICs of vancomycin and teicoplanin were not changed in any of the mutants. The MICs of cationic antimicrobial agents such as gentamicin and arbekacin were decreased in all the mutants, and the MICs of daptomycin and nisin A were decreased in the Δ*alr1* and Δ*cycA* mutants. The MICs of D-cycloserine, bacitracin, and lysostaphin were decreased in the Δ*alr1* and Δ*cycA* mutants. All the MIC changes in gene inactivation mutants were restored by gene complementation.

**TABLE 1:** MICs of several antimicrobial agents in MW2 WT and mutants^*a*^

MW2	OX	CEZ	VCM	TEIC	DAP	GM	ABK	Nisin A	DCS	BAC	Lysostaphin
WT	16	16	2	1	1	2	4	1,024	64	64	0.5
Δ*alr1*	1	4	2	1	0.5	1	0.5	512	32	32	0.125
*alr1*c	16	32	N.D.	N.D.	2	2	4	1,024	64	64	0.25
Δ*dat*	8	16	2	1	1	1	1	1,024	64	64	0.5
*dat*c	32	N.D.	N.D.	N.D.	N.D.	2	4	N.D.	N.D.	N.D.	N.D.
Δ*cycA*	1	4	2	1	0.5	1	1	512	32	32	0.25
*cycA*c	16	8	N.D.	N.D.	1	2	2	1,024	64	64	0.5

^*a*^ OX: oxacillin, CEZ: cefazolin, VCM: vancomycin, TEIC: teicoplanin, DAP: daptomycin, GM: gentamicin, ABK: arbekacin, DCS: D-cycloserine, BAC: bacitracin N.D., not determined.

**Fig 2:**
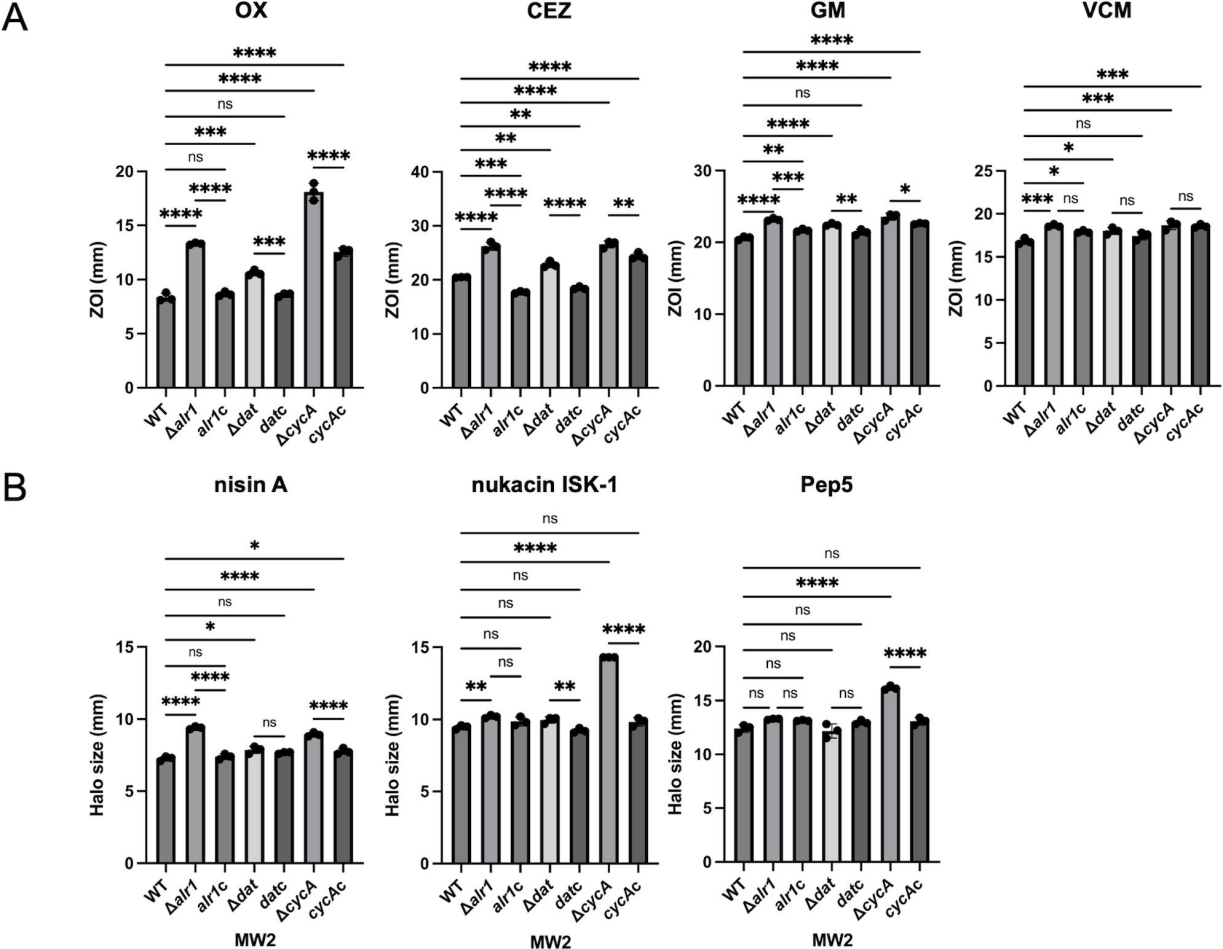
Results of the susceptibility tests in MW2 mutants. (**A**) Results of the disk tests with MW2 and its mutants for susceptibilities to oxacillin (OX), cefazolin (CEZ), gentamicin (GM), and vancomycin (VCM). The exact diameter of the zone of inhibition (ZOI, mm) was calculated from the mean value of three independent ZOIs. Statistical significance was determined by Tukey’s multiple comparison test. *P < 0.05; **P < 0.01; ***P < 0.001; ****P < 0.0001; ns, not significant. (**B**) Results of the direct assays with MW2 and its mutants for susceptibilities to nisin A, Pep5, and nukacin ISK-1. The exact diameter of the ZOI was calculated from the mean value of three independent ZOIs. Statistical significance was determined by Tukey’s multiple comparison test. *P < 0.05; **P < 0.01; ***P < 0.001; ****P < 0.0001; ns, not significant.

In the disk test, all the mutants presented increased susceptibility to oxacillin, cefazolin, and gentamicin; particularly, the significant changes were observed in the Δ*alr1* and Δ*cycA* mutants (Fig. 2A). Vancomycin susceptibility was slightly increased in the Δ*alr1,* Δ*dat,* and Δ*cycA* mutants, but this change was not restored in gene-complemented mutants. Susceptibility to cationic bacteriocins such as nisin A, Pep5, and nukacin ISK-1 was verified by a direct assay (Fig. 2B). The Δ*alr1* mutant presented increased susceptibility to nisin A, and the Δ*cycA* mutant presented increased susceptibility to all bacteriocins compared with the WT and complemented mutants. These mutants (Δ*alr1*, Δ*dat,* and Δ*cycA* mutants) showed slightly slower growth than the WT in trypticase soy broth (TSB), but there was no difference among the mutants (Fig. S1).

### The net surface negative charge is stronger in the Δ*alr1* and Δ*cycA* mutants

**Fig 3:**
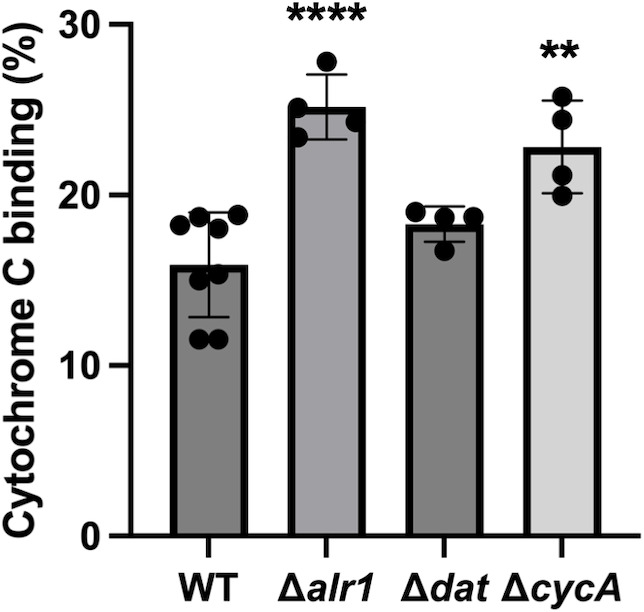
Cytochrome C binding ratio in MW2 mutants. The results of the cytochrome C binding assay using MW2 and its mutants in TSB. The binding ratio of cationic cytochrome C reflects the net negative charge of the cell surface. The exact cytochrome C binding ratio was calculated from the mean value of more than three independent experiments. Statistical significance was determined by Dunnett’s multiple comparison test. **P < 0.01; ****P < 0.0001.

Since the Dlt system weakens the net negative charge of *S. aureus* cells, we verified the net negative charge of each mutant by a cytochrome C binding assay. The amount of cytochrome C bound to bacterial cells significantly increased in the Δ*alr1* and Δ*cycA* mutants but did not significantly change in the Δ*dat* mutant (Fig. 3). These changes in the two mutants were restored by gene complementation (Fig. S2)

### Gene expression in the Δ*alr1*, Δ*dat*, and Δ*cycA* mutants

**Fig 4:**
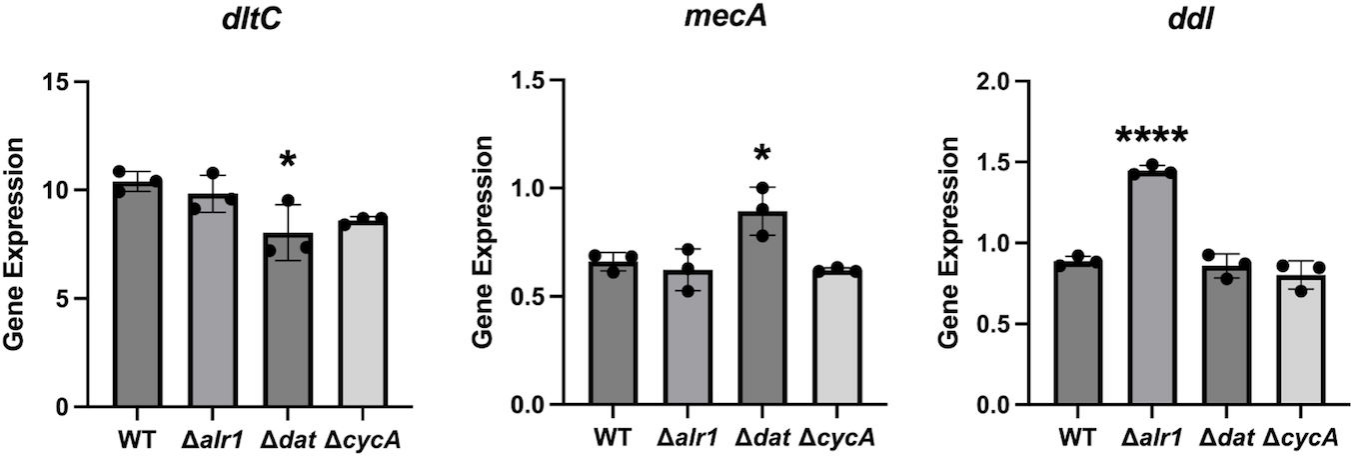
Gene expression of MW2 WT and its mutants. Gene expression of *dltC, mecA*, and *ddl* in MW2 WT and its mutants in TSB. The mean value was calculated from the data of three independent cDNA samples to determine the exact gene expression. Statistical significance was determined by Dunnett’s multiple comparison test. *P < 0.05; ****P < 0.0001.

We performed gene expression analysis to examine whether the changes in susceptibility and net surface charge are based on changes in gene expression. We selected the *dltC, ddl,* and *mecA* genes for analysis. *ddl* is the gene that encodes the enzyme D-alanine-D-alanine ligase, and the *mecA* gene encodes the methicillin resistance factor PBP2′. There were no significant changes in most of the data sets, but *dltC* expression was slightly decreased in the Δ*dat* mutant, *mecA* expression was slightly increased in the Δ*dat* mutant, and *ddl* expression was significantly increased in the Δ*alr1* mutant (Fig. 4).

### Cell wall thickness

Since cell wall thickness and hypocrosslinking of peptidoglycan are associated with the susceptibility to vancomycin and β-lactams (12, 13), we analyzed the thickness of the cell wall in MW2 WT and its mutants using transmission electron microscopy (TEM) images. No statistically significant changes were observed among WT, Δ*alr1*, Δ*dat*, and Δ*cycA* mutants (Fig. S3).

### The exogenous alanine concentration affects antimicrobial susceptibility

Our results revealed that the alanine racemase Alr1 and the alanine transporter CycA had relatively strong effects on antimicrobial susceptibility and cell surface charge. In *S. aureus*, alanine racemase is the main enzyme for D-alanine synthesis (6), but we speculated that the function of CycA might change depending on the amount of alanine in the environment. Mueller Hinton Broth (MHB) and TSB contain a digest of casein, and because casein contains alanine, it is expected that L-alanine was supplied in the previous experiments. Therefore, we used chemically defined medium (CDM) to verify the extent to which exogenous alanine affects antimicrobial susceptibility. We prepared CDM without L-alanine and with the glucose concentration restricted to 10 mM (designated CDM'G) and also prepared CDM’G with 10 mM L-alanine or D-alanine added to verify the susceptibility to several representative antimicrobial agents such as oxacillin, vancomycin, gentamicin, nisin A, D-cycloserine, and lysostaphin. In CDM′G, MICs to all tested antimicrobial agents were decreased compared with CDM’G with L- or D-alanine (Table 2). Furthermore, we analyzed the oxacillin and gentamicin susceptibility of Δ*alr1*, Δ*dat,* and Δ*cycA* mutants in CDM′G with or without alanine. Since the Δ*alr1* and Δ*dat* mutants grew slowly in CDM′G, the MICs were determined at 24 h and 48 h after inoculation. The Δ*alr1* and Δ*dat* mutants presented decreased oxacillin MICs in CDM′G with or without alanine (L- or D-alanine) compared with the WT strain (Table 3). In addition, the Δ*cycA* mutant presented decreased oxacillin MICs only in CDM′G with alanine (L- or D-alanine). The Δ*alr1* and Δ*dat* mutants had decreased gentamicin MICs compared with those of WT in CDM′G. However, the gentamicin MICs of Δ*alr1,* Δ*dat,* and Δ*cycA* were not different in CDM′G with L- or D-alanine, with the exception of the Δ*alr1* mutant after 48 h of incubation in CDM’G with D-alanine.

**TABLE 2:** MICs of several antimicrobial agents in CDM′G with or without alanine

MW2 WT	OX	VCM	GM	Nisin A	DCS	Lysostaphin
CDM′G	0.25	0.5	4	512	≤0.125	0.125
CDM′G + L-ala	4	1	8	1,024	128	0.5
CDM′G + D-ala	4	1	8	1,024	>128	0.5

**TABLE 3:** Oxacillin and gentamicin MICs of MW2 WT and mutants in CDM′G with or without alanine

	24 h			48 h		
	CDM′G	CDM′G +L-ala	CDM′G +D-ala	CDM′G	CDM′G +L-ala	CDM′G +D-ala
OX						
WT	0.25	4	4	1	32	16
Δ*alr1*	N.G.^*a*^	0.5	0.5	≤0.03125	2	1
Δ*dat*	N.G.	1	2	≤0.03125	4	4
Δ*cycA*	0.5	1	1	2	2	2
GM						
WT	4	8	8	8	16	16
Δ*alr1*	N.G.	8	8	4	16	8
Δ*dat*	N.G.	8	8	4	16	16
Δ*cycA*	4	8	8	8	16	16

^*a*^ N.G., no growth.

Since oxacillin susceptibility was drastically affected by alanine depletion, we investigated the relationship between oxacillin susceptibility and alanine concentrations. CDM′G containing 10 mM, 1 mM, 100 µM, or 10 µM L- or D-alanine was prepared, and the oxacillin MICs of MW2 WT and Δ*cycA* were verified in each medium. In the WT strain, the MIC was not decreased in CDM′G with 1 mM L-alanine compared to CDM′G with 10 mM L-alanine, but the MIC was decreased in CDM′G with 100 µM L-alanine or lower (Table 4). The Δ*cycA* mutant presented a decreased MIC compared with that of the WT in CDM′G with 10 mM, 1 mM, and 100 µM L-alanine and D-alanine.

**TABLE 4:** Oxacillin MICs of MW2 WT and Δ*cycA* mutant in CDM’G with several L- or D-alanine concentrations

	CDM′G + L-alanine		CDM′G + D-alanine	
conc.	WT	Δ*cycA*	WT	Δ*cycA*
10 mM	4	1	4	1
1 mM	4	1	8	2
100 µM	2	1	2	0.5
10 µM	0.25	0.5	0.25	0.5
0	0.25	0.5	0.25	0.5

### Gene expression in different media

**Fig 5:**
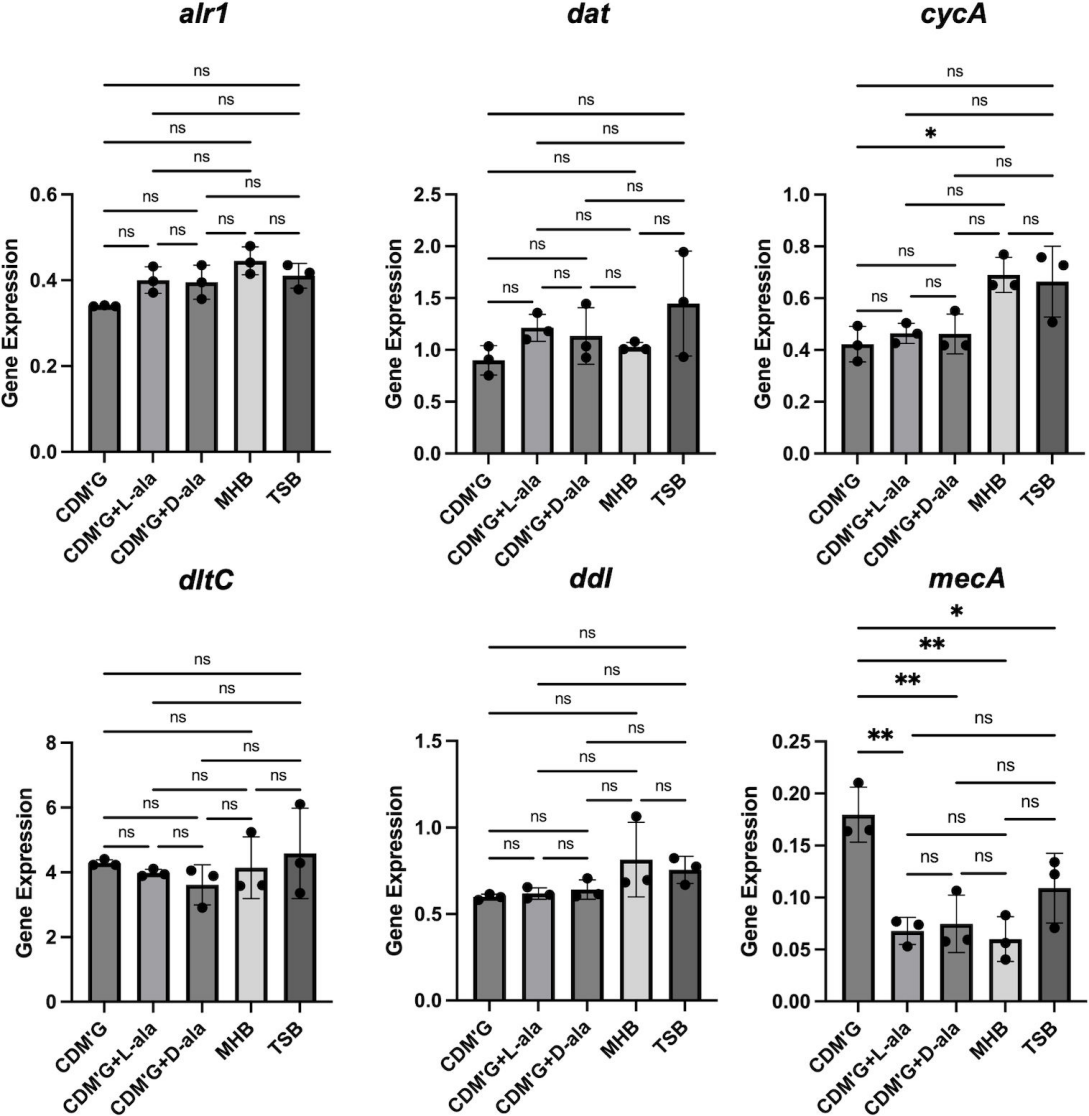
Gene expression of MW2 WT in CDM’G and CDM′G with L- or D-alanine. Gene expression of *alr1, dat, cycA, dltC, ddl*, and *mecA* in CDM′G, CDM′G with 10 mM L-alanine (CDM’G + L ala), CDM′G with 10 mM D-alanine (CDM’G + D ala), TSB, and MHB. The mean value was calculated from the data of three independent cDNA samples to determine the exact gene expression. Statistical significance was determined by Tukey’s multiple comparison test. *P < 0.05; **P < 0.01; ns, not significant.

The gene expression of *alr1, dat, cycA, dltC, ddl,* and *mecA* in MW2 WT was analyzed in CDM′G, CDM′G with 10 mM L- or D-alanine, MHB, and TSB (Fig. 5). Most of the data sets showed no significant changes, but the expression of *cycA* was lower in CDM’G than in MHB, and the expression of *mecA* was greater in CDM′G than in all other media.

### Clinical MRSA strains present increased susceptibility to oxacillin when alanine is absent from the medium

**Fig 6:**
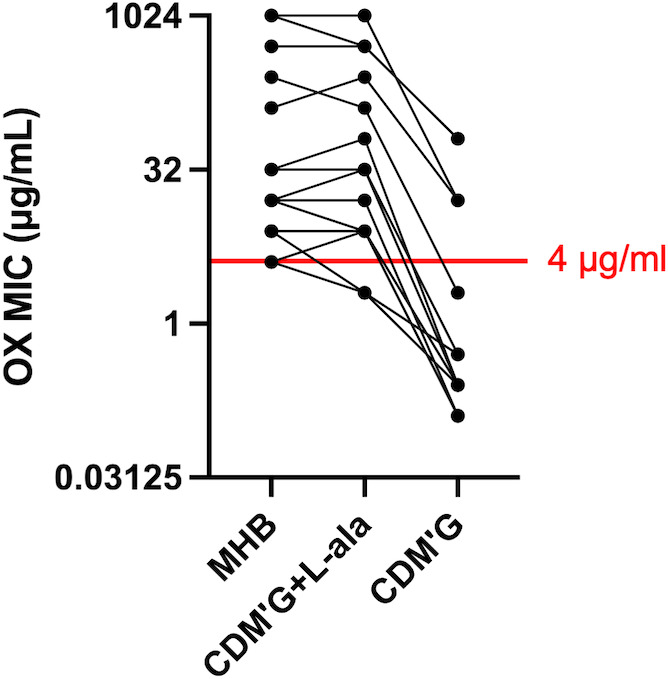
Oxacillin MICs of 16 clinical MRSA strains in MHB, CDM′G with 10 mM L-ala (CDM’G + L ala), and CDM′G. The points connected by lines indicate the MICs of the same strains. The criterion for resistance (MIC = 4 µg/mL or more) is shown as a red line. Strains with a MIC of 0.125 or less were plotted as having a MIC of 0.125. The exact MIC value was calculated from the median of three independent experiments.

Since alanine depletion strongly affects oxacillin susceptibility in MW2, we verified whether the same reduction was observed in 16 clinical isolates of MRSA. These MRSA strains were isolated from patients with bloodstream infections in Japan between 2019 and 2020 (14). These strains include famous MRSA lineages, such as the USA300 clone, the MRSA/J clone, and the New York Japan (NY/J) clone (Table S1). The oxacillin MICs in MHB, CDM′G, and CDM′G with the addition of 10 mM L-alanine are plotted in Fig. 6 (all MIC values are shown in Table S1). For all the MRSA strains, the MICs in CDM′G were reduced by 4–256 times compared with those in CDM′G containing 10 mM L-alanine. Although the MICs of most strains were below the criteria for resistance (MIC: 4 µg/mL or more) in CDM'G, which does not contain alanine, some highly resistant strains still exhibited oxacillin resistance in CDM′G. With respect to Newman, which is a methicillin-sensitive strain, the oxacillin MIC in CDM′G was four times lower than that in MHB and CDM′G with L-alanine added (Table S1).

## DISCUSSION

In this study, we revealed that inactivation of the factors involved in D-alanine synthesis or uptake systems increased susceptibility to β-lactams, other cell wall inhibitors, and cationic antimicrobial agents (Table 1 and Fig. 2).

β-Lactams are known to inhibit PBPs, which catalyze the crosslinking of peptidoglycan (transpeptidase activity) and/or extension of glycan chains (transglycosidase activity). Since the D-ala-D-ala moiety in peptidoglycan is cleaved by the carboxypeptidase activity of PBPs, leading to the formation of a glycine bridge between peptidoglycan chains by transpeptidase activity, the D-ala-D-ala structure is important for the activity of PBPs. Inhibition of D-alanine-D-alanine ligase (Ddl) is known to increase the number of muropeptides with a tripeptide stem in the cell wall, which, in turn, causes hypocrosslinking of the peptidoglycan chains (9, 15). It has been reported that the CycA-deficient mutant also presented increased tripeptide stems in the muropeptide, causing hypocrosslinking of peptidoglycan (9). Since approximately 80% of the intracellular D-ala-D-ala is derived from L-alanine taken up from the medium (6) and CycA is involved in alanine uptake, it is thought that the reduced amount of intracellular D-alanine causes hypocrosslinking of peptidoglycan in the Δ*cycA* mutant. It is predicted that the same phenomenon occurred in WT grown in CDM’G. Panda S et al. reported that Alr1 is mainly responsible for the synthesis of D-alanine in *S. aureus* and that the amount of D-alanine derived from Dat activity is lower (6). They also reported that the intracellular D-alanine pool and the crosslinking of peptidoglycan are decreased in the *alr1*-deficient mutant, whereas no significant difference was detected in the *dat-*deficient mutant (6). The slight increase in susceptibility to lysostaphin in the Δ*alr1* and Δ*cycA* mutants and the WT strain cultured in CDM’G also suggests the hypocrosslinking of peptidoglycan, as lysostaphin cleaves the pentaglycine bridge of peptidoglycan (Tables 1 and 2) (16). Hypocrosslinking of peptidoglycan is thought to confer increased β-lactam susceptibility, such as in *femA-* or *pbp4-*deficient mutants (17, 18), suggesting that the decreased intracellular D-alanine pool and peptidoglycan hypocrosslinking confer increased susceptibility to oxacillin and cefazolin in the Δ*alr1* and Δ*cycA* mutants, and also confer increased susceptibility to oxacillin in CDM′G (Tables 1 to 4 and Fig. 2). The gene expression of *mecA* was slightly increased in the Δ*dat* mutant but did not change in the Δ*alr1* and Δ*cycA* mutants (Fig. 4). The gene expression of *ddl* increased in Δ*alr1* but did not change in the other mutants (Fig. 4). The increased gene expression of *mecA* and *ddl* in Δ*alr1* and Δ*dat* mutants was not correlated with the susceptibility to oxacillin (Table 1 and Fig. 2A). In addition, the expression of *mecA* in WT grown in CDM’G was higher than that grown in other media (Fig. 5), but the MIC of oxacillin in CDM’G was not high compared to that of others (Table 2). These results suggest that the increased susceptibility to β-lactams in the Δ*alr1* and Δ*cycA* mutants and CDM′G is based on a decrease in the amount of intracellular D-alanine rather than on a change in the amount of enzyme, such as PBP2′.

Oxacillin MICs were lower in the Δ*alr1* and Δ*dat* mutants than in the WT in CDM’G with 10 mM L-alanine (Table 3). This is reasonable because the substrate of Alr is L-alanine, and the substrate of Dat is pyruvate, which is converted from L-alanine by alanine dehydrogenase (Fig. 1) (19). However, adding 10 mM D-alanine was also unable to compensate for the oxacillin susceptibility of the Δ*alr1* and Δ*dat* mutants compared with that of the WT. Since Alr can convert D-alanine to L-alanine and Dat can synthesize D-glutamate from D-alanine and α-ketoglutarate (20, 21), and L-alanine and D-glutamate are likely synthesized under D-alanine-sufficient conditions, we verified whether the shortage of L-alanine in the Δ*alr1* mutant and D-glutamate in the Δ*dat* mutant would result in increased susceptibility to oxacillin. The oxacillin MIC of the Δ*alr1* mutant was greater in CDM′G with 10 mM D-alanine and L-alanine than in CDM′G with only D-alanine (Table S2). In addition, the MIC of the Δ*dat* mutant was slightly increased in CDM′G with 10 mM D-alanine and D-glutamate. Since L-alanine and D-glutamate are also present in peptidoglycan, these amino acids may affect susceptibility to oxacillin by affecting the efficacy of peptidoglycan synthesis in addition to D-alanine (Fig. 1).

Since alanine depletion drastically increased oxacillin susceptibility in MW2, we verified whether other *S. aureus* strains, including clinical MRSA strains, also showed the same tendency. Compared with CDM′G with 10 mM L-alanine, all the tested strains were more susceptible to oxacillin in CDM′G (Fig. 6; Table S1). This result suggests that the effect of exogenous alanine on peptidoglycan synthesis is a universal phenomenon in *S. aureus*.

Hypocrosslinking of peptidoglycan and thickened cell wall is known as a common feature of vancomycin-intermediate *S. aureus* (13). It has been thought that the non-crosslinked D-ala-D-ala moiety becomes a pseudo-target for vancomycin, preventing it from reaching the peptidoglycan biosynthesis site on the cytoplasmic membrane and conferring resistance to vancomycin. However, from the previous observations of the *femC*-mutant BB589 strain, which showed the hypocrosslinking of peptidoglycan without cell wall thickening and normal susceptibility to vancomycin (22, 23), the hypocrosslinking is not always linked with cell wall thickness and the decreased vancomycin susceptibility (13). Our finding that Δ*alr1* and Δ*cycA* mutants are predicted to have hypocrosslinked peptidoglycan, but these mutants showed no difference in cell wall thickness and vancomycin MIC compared with WT (Fig. S3; Table 1), may correspond to the observation of the BB589 strain.

In addition to β-lactams, susceptibility to two cell wall inhibitors, bacitracin and D-cycloserine, was increased in the Δ*alr1* and Δ*cycA* mutants (Table 1). Since bacitracin inhibits peptidoglycan synthesis by inhibiting the recycling of lipid II (24), impaired peptidoglycan synthesis caused by a shortage of D-alanine may have a synergistic effect on the activity of bacitracin. Nisin A and nukacin ISK-1 also can inhibit lipid II (25, 26), and their activity may be affected in the same way as bacitracin, in addition to the effects of the cell surface charge described below.

D-cycloserine inhibits peptidoglycan synthesis by inhibiting Ddl and Alr (27). The amounts of L- and D-alanine, which are the substrates of these enzymes, may affect the activity of D-cycloserine. Consistent with this prediction, adding L- or D-alanine drastically increased D-cycloserine MIC in CDM'G (Table 2). This finding indicates that increased susceptibility to D-cycloserine is due to the decreased amount of intracellular alanine in the Δ*alr1* and Δ*cycA* mutants.

Teichoic acid modification with D-alanine is known to affect the net negative charge of the cell surface, resulting in decreased susceptibility to cationic antimicrobial agents such as gentamicin, daptomycin, vancomycin, nisin, Pep5, and nukacin ISK-1 (4, 28–32). This modification is completely dependent on the Dlt system, which is composed of four proteins, DltABCD (4). This system uses intracellular D-alanine to esterify the phosphate group of teichoic acids (33). Therefore, we predicted that the amount of intracellular D-alanine affects the efficacy of this system and susceptibility to cationic antimicrobial agents. The susceptibility to gentamicin and arbekacin was increased in the Δ*alr1,* Δ*dat,* and Δ*cycA* mutants, the susceptibility to daptomycin and nisin A was increased in the Δ*alr1* and Δ*cycA* mutants, and the susceptibility to nukacin ISK-1 and Pep5 was increased in the Δ*cycA* mutant (Table 1 and Fig. 2). A cytochrome C binding assay revealed an increased net negative charge in the Δ*alr1* and Δ*cycA* mutants (Fig. 3). The *dltC* expression was slightly decreased in the Δ*dat* mutant, whereas no changes were observed in other mutants (Fig. 4). Increased susceptibility to gentamicin and nisin A and increased net negative charge were observed in WT grown in CDM’G compared with the WT grown in CDM′G with 10 mM L- or D-alanine, whereas the MIC of gentamicin in the Δ*dltA* mutant was not altered in CDM′G and CDM′G with alanine (Table 2; Fig. S4 and Table S3). These findings suggest that Dlt-mediated teichoic acid modification and subsequent alteration of cell surface charge are affected by the restriction of the D-alanine or L-alanine supply, resulting in increased susceptibility to cationic antimicrobial agents.

Our study indicates the possibility of a potential synergistic effect between D-alanine supply inhibitors and several types of antimicrobial agents. At this point, D-cycloserine is known to have a synergistic effect on β-lactams, daptomycin, and vancomycin (9, 34, 35). In this study, we found that not only D-alanine biosynthesis but also the uptake of exogenous alanine significantly affects the susceptibility to antimicrobial agents. These findings suggest that drugs that inhibit the uptake of alanine could also have a synergistic effect. Although inhibitors of amino acid transporters have been investigated as anticancer agents (36), none of these are known antimicrobial agents; thus, their development is expected in the future.

In conclusion, we revealed the effects of D-alanine synthesis and exogenous alanine on the antimicrobial susceptibility of *S. aureus*. We hope that this study contributes not only to the basic understanding of *S. aureus* but also to the consideration of treatment methods for *S. aureus* infections.

## MATERIALS AND METHODS

### Bacterial strains and growth conditions

The bacterial strains used in this study are shown in Table 5.

**TABLE 5:** Bacterial strains used in this study^*a*^

Strain	Characteristics	Reference
*Staphylococcus aureus*		
RN4220	NCTC8325-4 restriction- methylation+	(37)
MW2	USA400 lineage clinical MRSA	(11)
MW2 Δ*alr1*	*alr1* inactivation in MW2, Tc^r^	This study
MW2 *alr1* complement (*alr1*c)	*alr1* complemented in MW2 *alr1*-inactivated mutant, Tc^r^, Cp^r^	This study
MW2 Δ*dat*	*dat* inactivation in MW2, Tc^r^	This study
MW2 *dat* complement (*dat*c)	*dat* complemented in MW2 *dat-*inactivated mutant, Tc^r^, Cp^r^	This study
MW2 Δ*cycA*	*cycA* inactivation in MW2, Tc^r^	This study
MW2 *cycA* complement (*cycA*c)	*cycA* complemented in MW2 *cycA-*inactivated mutant, Tc^r^, Cp^r^	This study
Clinical MRSA strains	Clinical MRSA strains isolated from bloodstream infection	(14)
Newman	MSSA standard strain ATCC 25904	(38)
MW2 Δ*dltA*	*dltA* knockout in MW2, Em^r^	This study
Others		
*Escherichia coli* XL-II	Competent cell for transformation	(31)
*Staphylococcus epidermidis* KSE112	Pep5 producer	(31)
*Staphylococcus warneri* ISK-1	Nukacin ISK-1 producer	(39)
*Lactococcus lactis* ATCC11454	Nisin A producer	(40)

^*a*^ Tc^r^: tetracycline resistance, Cp^r^: chloramphenicol resistance, Em^r^: erythromycin resistance.

*Staphylococcus* strains were grown in TSB (Becton, Dickinson and Company [BD], Franklin Lakes, NJ, USA) at 37°C with shaking. *Lactococcus lactis* strain was grown in TSB at 37°C with static incubation in a 5% CO_2_ incubator. When necessary, 5 µg/mL tetracycline (Wako Pure Chemical Corporation, Osaka, Japan), 10 µg/mL chloramphenicol (Wako Pure Chemical Corporation), or 10 µg/mL erythromycin (Wako Pure Chemical Corporation) was added to the TSB for *S. aureus* mutants, and 100 µg/mL ampicillin (Nacalai Tesque, Inc., Kyoto, Japan) was added for *E. coli*.

### Chemically defined medium

The method for preparing CDM was described previously (41). To investigate the effects of alanine, we created a CDM that did not contain L-alanine and restricted the final concentration of glucose to 10 mM (designated as CDM′G). When necessary, 10 mM L-alanine (Katayama Kagaku Industries, Osaka, Japan), D-alanine (Katayama Kagaku Industries), and/or D-glutamate (Tokyo Chemical Industry, Tokyo, Japan) was added to CDM′G.

### Construction of *S. aureus* genetic mutants

Gene inactivation and knockout in *S. aureus* MW2 were performed with the thermosensitive plasmid pYT1 as described previously (31). Genetic complementation was performed with the plasmid pCL8, a derivative of pLI50 (42). For the complementation of *alr1* and *cycA*, a single fragment containing the upstream promoter region and gene-coding region was created by PCR. For the complementation of *dat,* the fragments containing the upstream promoter region and the *dat*-coding region were created separately because there was another gene (*pepV*) between the *dat*-coding region and its promoter. These fragments were subsequently fused by overwrapping PCR. The obtained fragments were inserted and cloned into pCL8 and introduced into MW2 mutants as described previously (31). The primers used in this method are shown in Table 6 (for creating nucleotide fragments and checking the insertion of pYT1 into the target gene or genetic replacement).

**TABLE 6:** Primers used in this study

Target gene	Forward primer (5′→3′)	Reverse primer (5′→3′)
Used for quantitative PCR		
*alr1*	GGTCGCGTATGTATGGATCA	ACCACCTCTACCGACTGTGG
*dat*	TGGTGTGAATGGTGTTACCG	GACAGTTTCGCCTCGATGTT
*cycA*	CTGGGGAAACAAAAGATCCA	AACGGAATACCGATCAATGC
*dltC*	AGCAGAAGTAGCAGAAAATG	GCCCACTCATCTCTATCAA
*ddl*	GGCCAGGTGAAGTCGTAAAA	TGTCGCTTTGAATGCCTCTA
*mecA*	ATTGGGATCATAGCGTCA	ATCTCATATGCTGTTCCTGT
*gyrB*	AGGTCTTGGAGAAATGAATG	CAAATGTTTGGTCCGGTT
Used for constructing *S. aureus* gene inactivated and KO mutants		
*dltA*-UP	ATGGATCCATAAATATGTTGAAGCATTC	CAGTCGAGGATATAATCATTTTAATACGGTC
*dltA-*DOWN	GCTGACCTAGTAATTGACAGAAAGAAAATTG	ATAAGCTTTTAATTTACTTTCATGGAAG
*alr1*	ATGGATCCTTATAGATCTGCGTATATGA	ATAAGCTTATACAAGTTGCTCATATTG
*dat*	ATGGATCCAATGGTGAGTTTGTAAGTC	TCAAGCTTTTAATGTCGCAA
*cycA*	ATGGATCCACATCATTATAGGATTTATG	ATAAGCTTCATATAAATTTGTGAAACTG
Used for checking the insertion of pYT1 into target genes		
*alr1*	ACAATGATAATTAAGATCTAG	CACAGGAAACAGCTATGACCATG
*dat*	AAGCAATTTATTCATTATGC	CACAGGAAACAGCTATGACCATG
*cycA*	AAATTACAAAGGGAACTGA	CACAGGAAACAGCTATGACCATG
Used for checking gene replacement		
*dltA*::*em^r^*	AAATCTAAAAGTAAACAGCC	ACTAGGTCAGCTTATTTCCTCCCGTTAAA
Used for constructing gene-complemented mutants		
*alr1*	ATAAGCTTCATGAGCAACGTAAAATTG	ATGGATCCATGAACTTTAATTACTCTAATG
*dat*-promoter	ATGTCGACAGTAGGACAGAAATGATAAG	ATTTTTTCCATTCGAAATCGACTTCCTT
*dat*	TCGATTTCGAATGGAAAAAATTTTTTTAAATG	ATGGATCCTAATTCACTTACGAAAGTTG
*cycA*	ATGATATCGCCACAAATAGCACCATTAA	ATGGATCCACTTTAGTATATTCAACAGG

### Determination of the MIC

The MICs of the antimicrobial agents were determined by the microdilution method described previously (31). MHB (BD) or CDM was used as the growth medium. For the CDM assay, bacterial cells were collected by centrifugation from the overnight culture, washed with PBS, and then collected by centrifugation again and suspended in CDM′G. The resulting suspension was diluted 100-fold and used in the susceptibility test. Oxacillin (OX), teicoplanin (TEIC), nisin A, and bacitracin (BAC) were purchased from Sigma-Aldrich, St. Louis, MO, USA. Vancomycin (VCM) and lysostaphin were purchased from Wako Pure Chemical Corporation. Daptomycin (DAP) and D-cycloserine (DCS) were purchased from Tokyo Chemical Industry. Arbekacin (ABK) was purchased from Meiji Seika Kaisha, Ltd., Tokyo, Japan. Gentamicin (GM) was purchased from Nacalai Tesque, Inc. For daptomycin, 50 µg/mL Ca^++^ was added to the medium. For lysostaphin, 0.1% bovine serum albumin was added to the medium. The assay was performed at least three times independently, and the median was used as the exact MIC value.

### Disk test

To evaluate antimicrobial susceptibility, we performed a disk test for four antimicrobial agents: oxacillin, cefazolin, gentamicin, and vancomycin. All disks were purchased from Eiken Chemical Co., Ltd., Tochigi, Japan. The overnight cultures of the indicator strains were adjusted to an optical density at 660 nm of 1.0 (1 × 10^9^ cells/mL). Prewarmed MHB soft agar (3.5 mL, 0.8% agar) containing 10^7^ indicator cells (adding 10 µL of a culture with an OD_660_ = 1.0) was poured onto Mueller Hinton agar (15 mL, 2% agar) and allowed to cool. The disks were subsequently placed on the soft agar. After incubation for 24 h at 37°C, the diameter of the inhibition area was measured. The exact data were calculated from the mean value of three independent inhibition areas.

### Direct assay

To evaluate bacteriocin susceptibility, we performed a direct assay as described previously (31). *L. lactis* ATCC11454 was used as a nisin A producer, *Staphylococcus epidermidis* KSE112 was used as a Pep5 producer, and *Staphylococcus warneri* ISK-1 was used as a nukacin ISK-1 producer (Table 5). The exact data were calculated from the mean value of three independent inhibition areas.

### Cytochrome C binding assay

To evaluate the cell surface charge, we performed a cytochrome C binding assay modified from a previous method (43). Cytochrome C was purchased from Sigma Aldrich. A total of 10^8^ *S. aureus* cells were inoculated into 10 mL of TSB or CDM and grown at 37°C with shaking. When the OD_660_ reached 1.0, 10^10^ cells were collected by centrifugation. After being washed with 10 mM sodium-phosphate buffer (pH 6.8) two times, the cells were suspended in 1 mL of buffer containing 400 µg of cytochrome C and exposed for 10 min at room temperature. After centrifugation, the absorbance of the supernatant at 530 nm was measured with a SpectraMax iD3 (Molecular Devices, San Jose, CA, USA). The absorbance values were compared with those of samples without bacterial cells to calculate the absorption ratio, which reflects the cell surface charge. At least three independent experiments were performed, and the average binding ratio was calculated.

### Quantitative PCR

Quantitative PCR was performed to evaluate gene expression. The cells for analysis were collected when the OD_660_ reached 0.58–0.66. RNA extraction, cDNA synthesis, and quantitative PCR were performed as described previously (31). Gene expression levels were calculated as the ratio of total *gyrB* expression. The primers used in this assay are listed in Table 6. cDNA samples were obtained from three independent samples, and the mean value was determined as the exact gene expression level.

### Measurement of growth curves

The overnight cultures of MW2 and its mutants grown in TSB were adjusted to an OD_660_ of 1.0, and the cells were collected by centrifugation and resuspended in fresh TSB. Aliquots of this suspension (10^8^ cells) were inoculated into 5 mL of fresh TSB, and the growth of each strain was monitored by measuring the OD_660_. The three independent tubes were used for analysis, and the mean values are shown as exact growth curves.

### Measurement of cell wall thickness

Measurement of cell wall thickness was performed using TEM images. Bacterial cells from the overnight culture of MW2 WT and its mutants were collected by centrifugation, washed with PBS, and then collected by centrifugation again and suspended in fresh TSB. Aliquots of this suspension (10^8^ cells) were inoculated into 12 mL of fresh TSB and grown at 37°C with shaking. When the OD_660_ reached 0.77–0.88, the bacterial cells were collected by centrifugation and suspended in 1.4 mL of 0.1 M phosphate buffer (pH 7.3) with 2.5% glutaraldehyde (Wako Pure Chemical Corporation). After fixing the samples at room temperature for 1 h, the samples were kept at 4°C and washed four times with 0.1 M phosphate buffer (pH 7.4) and subsequently post-fixed with 1.5% OsO_4_ for 2 h on ice. After the fixed cells were washed three times with 0.1 M phosphate buffer (pH 7.4), the samples were dehydrated by passing samples through an ethanol series (30%, 50%, 70%, 90%, and 100%, 20 min each). The obtained samples were embedded in the epoxy resin (Spurr Low Viscosity Embedding Kit, Polysciences, Inc., PA, USA), and the resin was polymerized at 70°C for 12 h. For TEM observation, ultrathin sections (70 nm) were obtained with an ultra-microtome UC7 (Leica Microsystems CMS GmbH, Germany) using a diamond knife. The ultrathin sections were stained with 3% uranyl acetate for 10 min and with lead stain solution (Sigma-Aldrich) for 3 min. TEM images were obtained using a JEM-1400 Plus (JEOL, Tokyo, Japan) with 80 kV of acceleration voltage.

The thickness of the cell wall in each image was quantified by comparing the thickness of the cell wall to the length of the scale bar. The exact thickness of the cell wall was calculated from ten independent cells.

### Statistical analysis

Dunnett’s multiple comparison test was performed for the comparison of cytochrome C binding ratios in Fig. 3, gene expression in Fig. 4, and cell wall thickness in Fig. S3. Tukey’s multiple comparison test was performed for the comparison of susceptibilities in Fig. 2, gene expression in Fig. 5, and cytochrome C binding ratios in Fig. S2 and S4. All the statistical analyses were performed with GraphPad Prism (GraphPad Software, San Diego, CA, USA).

## SUPPLEMENTAL MATERIAL

Supplemental material aac.01936-24-s0001.pdf Table S1 to S3; Fig. S1 to S4.

ASM does not own the copyrights to Supplemental Material that may be linked to, or accessed through, an article. The authors have granted ASM a non-exclusive, world-wide license to publish the Supplemental Material files. Please contact the corresponding author directly for reuse.
