# Supplementary material for: D-alanine synthesis and exogenous alanine affect the antimicrobial susceptibility of Staphylococcus aureus
Source: Antimicrob Agents Chemother. 2025 Jun 12;69(7):e01936-24. doi: 10.1128/aac.01936-24 (PMC12217452; doi:10.1128/aac.01936-24)
Supplement: Supplemental material — Table S1 to S3; Fig. S1 to S4. [file aac.01936-24-s0001.pdf]

Strain	CDM’G	CDM'G+L-ala	MHB	MLST	clone
**MRSA**					
10019-19-S014	64	512	512	764	
12004-19-S001	0.25	64	32	8	USA300 clone
17005-19-S001	0.25	32	16	8	MRSA/J clone
17005-19-S017	64	512	1024	5	NY/J clone
08024-19-S002	0.25	8	16	1	
22026-19-S002	2	128	256	5	NY/J clone
01040-19-S002	16	256	128	764	
01040-19-S024	≤0.125	8	8	1	
01040-19-S028	0.5	32	32	8	USA300 clone
12023-19-S007	16	1024	1024	5	NY/J clone
21016-19-S004	≤0.125	8	8	2725	
24001-19-S013	0.25	8	16	2725	
10019-19-S042	≤0.125	8	4	8	MRSA/J clone
12004-19-S012	≤0.125	16	16	1	
15029-19-S001	0.5	2	8	2725	
33009-19-S058	0.25	2	4	121	
**MSSA**					
Newman	0.0625	0.25	0.25	254	

Table S1. Oxacillin MICs of clinical MRSA strains and Newman in CDM’G and CDM’G with 10 mM L-alanine.

CDM’G+L-ala: CDM’G with 10 mM L-alanine, MLST: Multi-Locus Sequence Type
The MICs were measured after 24 h incubation.

MW2	CDM’G +D-ala	CDM’G +D-ala +L-ala	CDM’G +D-ala +D-glu
WT	2	2	2
Δ*alr1*	0.25	1	0.125
Δ*dat*	1	1	2

Table S2. The oxacillin MICs of MW2 WT, Δ*alr1,* and Δ*dat* mutants in CDM’G with or without several amino acids.

CDM’G+D-ala: CDM’G with 10 mM D-alanine, CDM’G+D-ala+L-ala: CDM’G with 10 mM D-alanine and 10 mM L-alanine
CDM’G+D-ala+D-glu: CDM’G with 10 mM D-alanine and 10 mM D-glutamate
The MICs were measured after 24 h incubation.

MW2	CDM'G	CDM'G+L-ala	CDM'G+D-ala	MHB
WT	4	8	8	2
Δ*dltA*	1	1	1	0.25

Table S3. The gentamicin MICs of MW2 and Δ*dltA* mutant.

CDM’G+D-ala: CDM’G with 10 mM D-alanine, CDM’G+L-ala: CDM’G with 10 mM L-alanine
The MICs were measured after 24 h incubation.

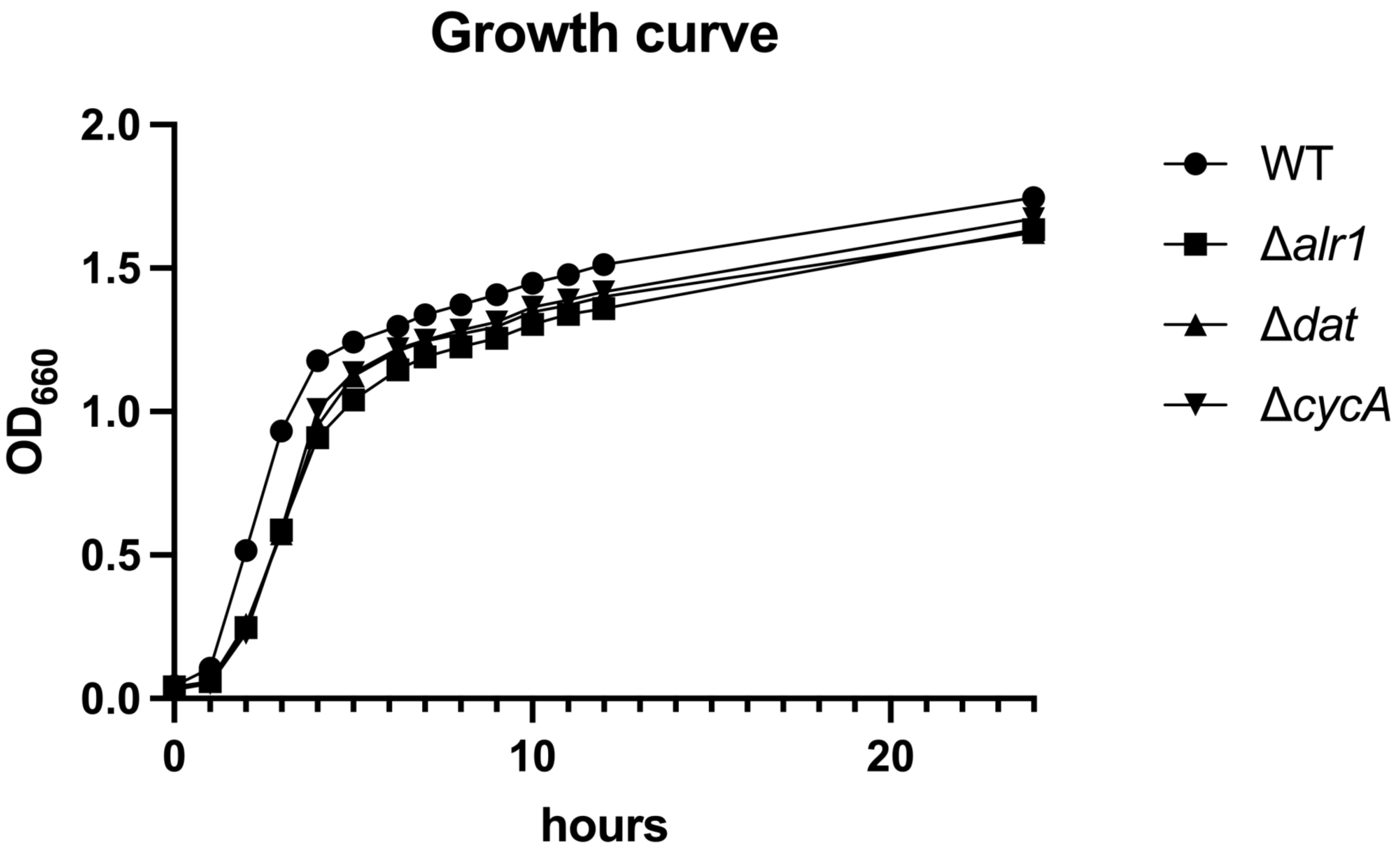

Fig. S1 Growth curves of MW2 WT, Δ*alr1*, Δ*dat* and Δ*cycA* mutants in TSB.

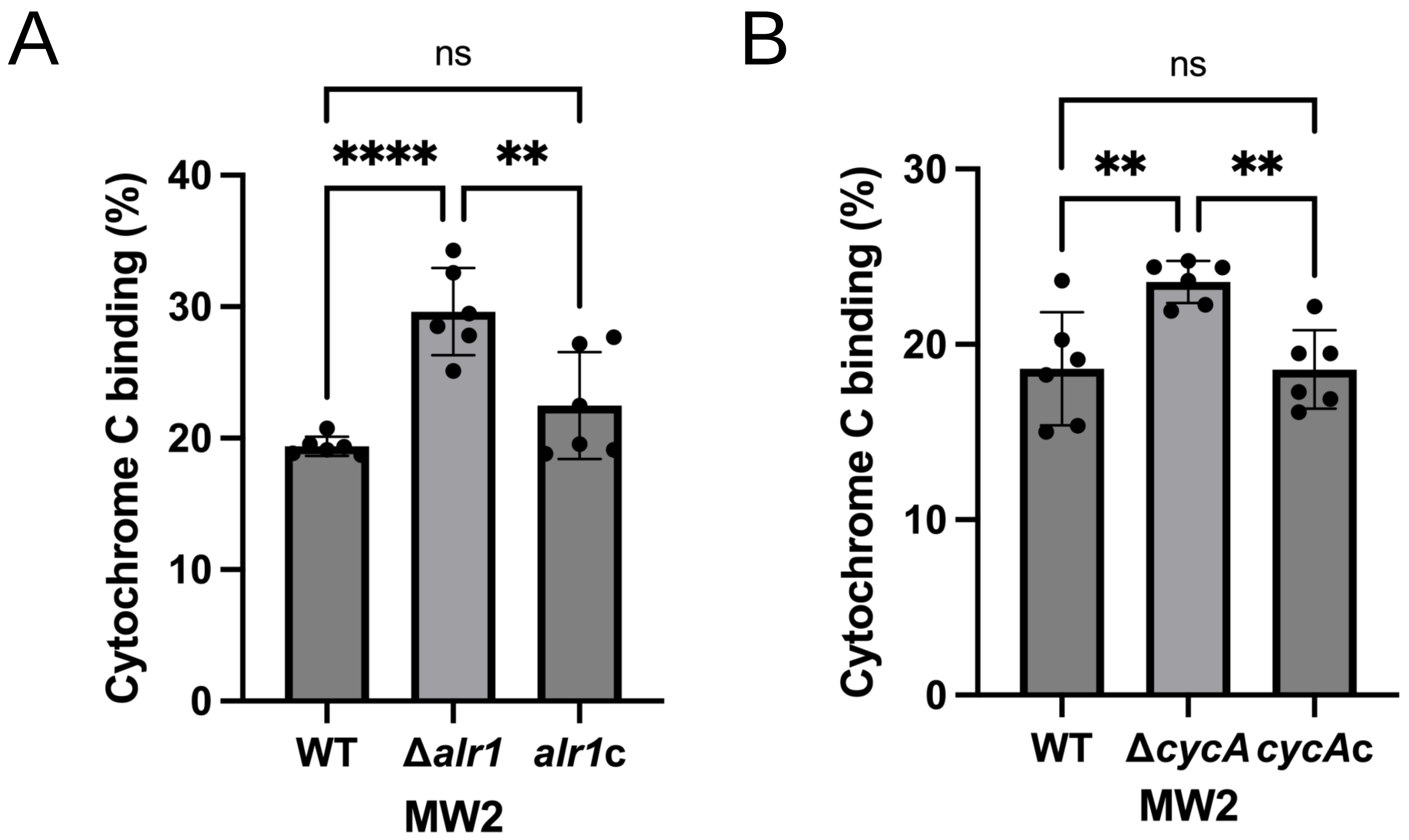

Fig. S2 Cytochrome C binding assay in MW2 genetically complemented mutants.

The results of the cytochrome C binding assay using MW2 WT, Δ*alr1*, *alr1*-complemented mutant (A), Δ*cycA* and *cycA*-complemented mutant (B) in TSB. The exact cytochrome C binding ratio was calculated from the mean value of six independent experiments. Statistical significance was determined by Tukey's multiple comparison test. **, $P < 0.01$; ****, $P < 0.0001$; ns, not significant.

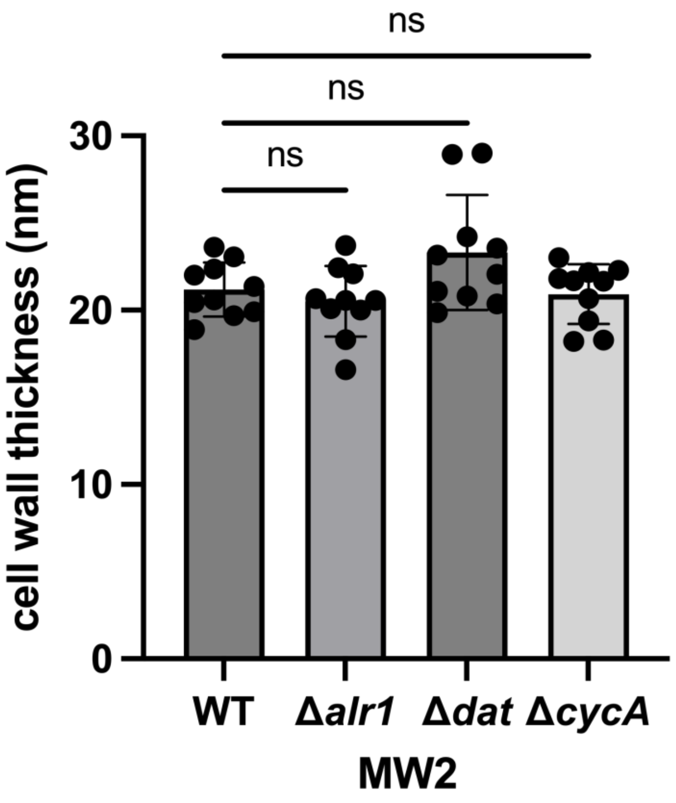

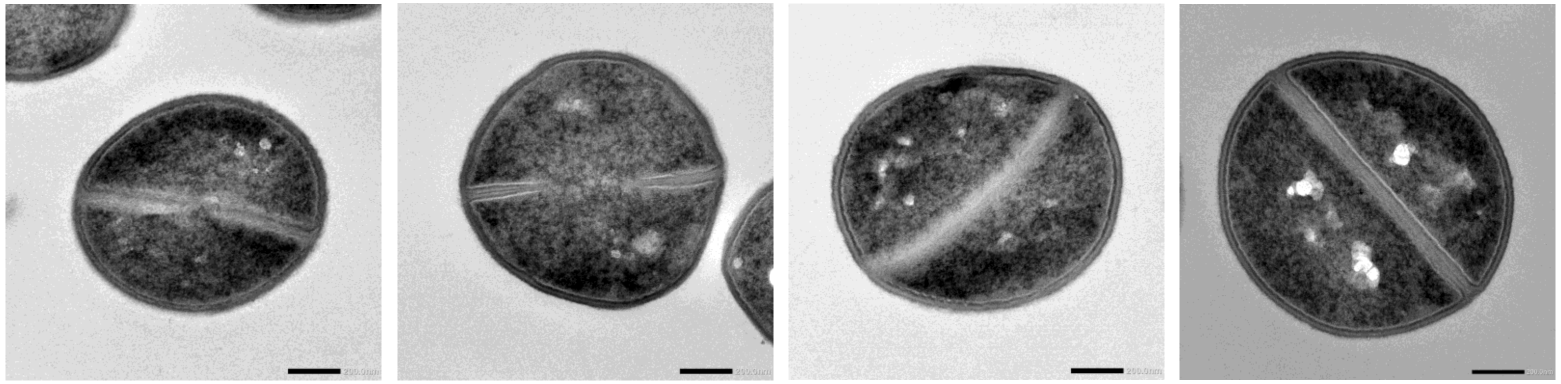

MW2 WT
21.2 ± 1.5 nm

Δ*alr1*
20.5 ± 1.9 nm

Δ*dat*
23.3 ± 3.1 nm

Δ*cycA*
20.9 ± 1.6 nm

Fig. S3 Cell wall thickness in MW2 WT and its mutants.

The comparison of cell wall thickness in MW2 WT, Δ*alr1,* Δ*dat,* Δ*cycA* mutants in TSB. Black scale bars represent 200 nm. The cell wall thickness of each sample was measured from the TEM images of ten independent cells. Statistical significance was determined by Dunnett's multiple comparison test. ns, not significant.

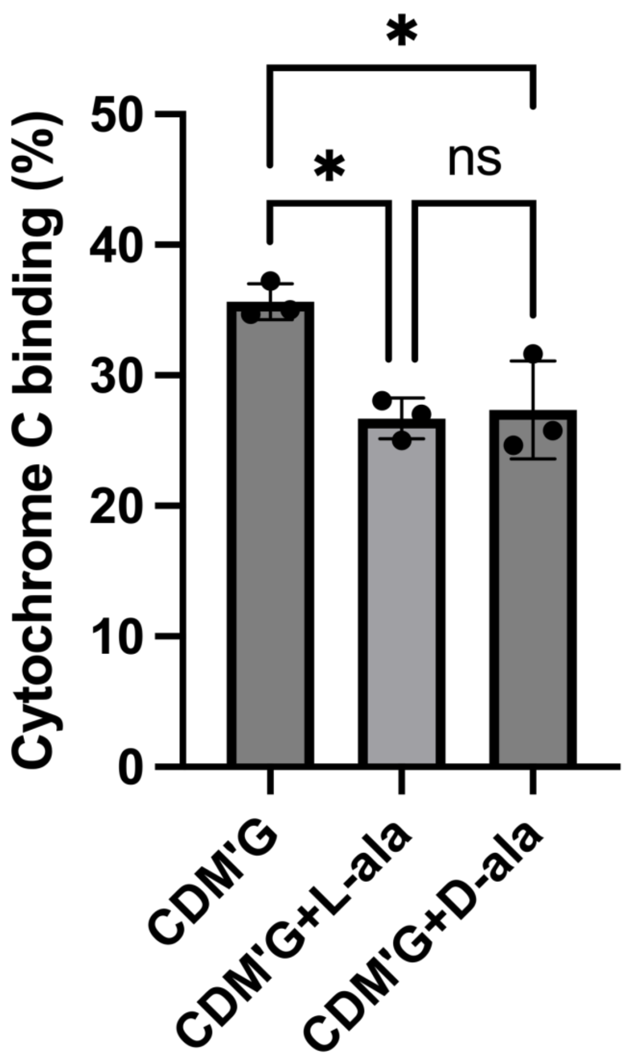

Fig. S4 Cytochrome C binding assay in CDM’G with or without alanine.

The results of the cytochrome C binding assay using MW2 WT. The exact cytochrome C binding ratio was calculated from the mean value of three independent experiments. Statistical significance was determined by Tukey’s multiple comparison test. *, $P < 0.05$; ns, not significant.
